# Sensing and Control Strategies Used in FES Systems Aimed at Assistance and Rehabilitation of Foot Drop: A Systematic Literature Review

**DOI:** 10.3390/jpm14080874

**Published:** 2024-08-17

**Authors:** Estefanía González-Graniel, Jorge A. Mercado-Gutierrez, Saúl Martínez-Díaz, Iliana Castro-Liera, Israel M. Santillan-Mendez, Oscar Yanez-Suarez, Ivett Quiñones-Uriostegui, Gerardo Rodríguez-Reyes

**Affiliations:** 1 División de estudios de Posgrado e Investiagación, TecNM-Instituto Tecnológico de la Paz, La Paz 28080, Mexico; m17310162@lapaz.tecnm.mx (E.G.-G.); iliana.cl@lapaz.tecnm.mx (I.C.-L.); israel.sm@lapaz.tecnm.mx (I.M.S.-M.); 2 Instituto Nacional de Rehabilitación Luis Guillermo Ibarra Ibarra, Mexico City 14389, Mexico; jmercado@inr.gob.mx (J.A.M.-G.); iquinones@inr.gob.mx (I.Q.-U.); 3 Electrical Engineering Department, Universidad Autónoma Metropolitana—Unidad Iztapalapa, Mexico City 09340, Mexico; oyanez@izt.uam.mx

**Keywords:** stroke, functional electrical stimulation, sensing, inertial measurement unit, EMG, rehabilitation, vision systems, foot drop

## Abstract

Functional electrical stimulation (FES) is a rehabilitation and assistive technique used for stroke survivors. FES systems mainly consist of sensors, a control algorithm, and a stimulation unit. However, there is a critical need to reassess sensing and control techniques in FES systems to enhance their efficiency. This SLR was carried out following the PRISMA 2020 statement. Four databases (PubMed, Scopus, Web of Science, Wiley Online Library) from 2010 to 2024 were searched using terms related to sensing and control strategies in FES systems. A total of 322 articles were chosen in the first stage, while only 60 of them remained after the final filtering stage. This systematic review mainly focused on sensor techniques and control strategies to deliver FES. The most commonly used sensors reported were inertial measurement units (IMUs), 45% (27); biopotential electrodes, 36.7% (22); vision-based systems, 18.3% (11); and switches, 18.3% (11). The control strategy most reported is closed-loop; however, most of the current commercial FES systems employ open-loop strategies due to their simplicity. Three main factors were identified that should be considered when choosing a sensor for gait-oriented FES systems: wearability, accuracy, and affordability. We believe that the combination of computer vision systems with artificial intelligence-based control algorithms can contribute to the development of minimally invasive and personalized FES systems for the gait rehabilitation of patients with FDS.

## 1. Introduction

The study of targeted rehabilitation has become an important aspect of stroke management. Stroke is a major cause of severe and long-term disability, often resulting in foot drop (FD), an impairment seen in 20% to 30% of stroke survivors. [1,2]. FD can result in gait related deficiencies. Is characterized by weakness or loss of control in the ankle and toe dorsiflexor muscles. Many survivors are permanently disabled and usually need physical therapy to regain their daily living abilities and prevent further loss of their remaining voluntary functions [1,3,4]. FD can arise from a variety of conditions, including muscular, neurological, spinal, autoimmune, and musculoskeletal disorders; these conditions frequently follow traumatic events such as vehicle accidents, sports and recreational activities, and medical procedures such as lumbosacral spine and hip replacement surgeries, among others [5,6].

However, survivors still retain excitable peripheral nerves and muscle tissues that may be re-established through functional electrical stimulation (FES) [7]. This method bypasses the biological lesion and delivers the necessary stimulation to induce muscle contractions [7]. FES is a rehabilitation tool for restoring motor skills in stroke survivors. By delivering electrical impulses through the skin and targeting specific muscles and nerves, it enables movements involved in specific functional tasks. This method is widely employed to help neurologically impaired individuals restore walking ability [8]. The aim of FES intervention is to enhance gait function by either substituting or assisting voluntary movements to achieve the desired motion [8,9]. Nevertheless, significant challenges remain despite ongoing advancements. A key issue lies in the structure of control strategies of neuroprostheses, which need to synchronize muscle movements with sensory feedback integration [8].

FES is a complex and dynamic process influenced by muscle fatigue, patient effort, and spasticity, which can vary unpredictably during treatment. Minor variations in electrode placement from day to day can significantly alter motor responses. To achieve a more robust FES system, it is essential to measure human motion and muscle contraction via lightweight, portable, and real-time-capable measurement systems [7].

Building on this understanding, in the following sections, two key blocks of FES systems are explored, sensor techniques and control strategies, in order to present an overview of which are used today, their limitations, and the advantages.

### 1.1. Sensing Techniques

FES systems consist mainly of sensors, a control algorithm, and a stimulation unit. Sensors offer crucial feedback to FES systems, allowing the control system to adjust stimulation outputs based on changes in parameters and environmental interactions [6]. Therefore, feedback sensors are a key element in the design of FES systems for the gait rehabilitation of patients with foot drop. Hence, having a quick reference of the state of the art in this topic would be of great help for new and potential researchers in the field. Such a reference would save them plenty of time, work, and resources, before testing different combinations. This systematic literature review (SLR) is focused on these topics, with the main aim of serving as a first reference and guide to FES researchers and designers, before embarking in technology development or clinical trials.

Wearable sensors, such as foot pressure insoles, foot switches, accelerometers, gyroscopes, inertial measurement units (IMUs), and electromyography (EMG) electrodes, have been extensively utilized in FES control strategies [8]. Particularly, IMUs have been used extensively in this field, since they integrate gyro-scopes, accelerometers, and magnetometers to estimate joint angles, gait, and angular velocities through sensor fusion techniques. These combined measures enable detailed motion monitoring in gait-oriented FES applications, enabling access to specific kinematic variables in real-time, like acceleration and orientation of relevant body segments, such as the foot, ankle, and lower and upper leg. The accuracy and precision of wearable sensing systems are influenced by the number and placement of sensors, their alignment with the body’s coordinate system, and the signal processing algorithms employed. Moreover, effective calibration methods in FES systems depend on sensors, such as non-restrictive sensor-to-segment calibration or automatic anatomical calibration, and are crucial for obtaining precise measurements and reliable data [10,11,12].

Muscle activity, specifically the recruitment of motor units, can be monitored using surface electromyography (sEMG). The sEMG signal represents the activity muscle in the form of a voltage that can be measured by surface electrodes and is closely related to the timing and intensity of muscle contraction [10]. sEMG signals are less favored in wearable gait, such as those based on FES, due to their complexity in acquisition and post-processing. Nevertheless, the sEMG signals evoked by electrical stimulation can be employed to predict the resultant joint torque, which provides a necessary prediction of the muscle response before achieving accurate joint torque controlled by FES [8].

Vision-based non-wearable technologies utilizing standard or depth cameras are increasingly used for tracking human motion. It has been reported recently that these cameras can extract the detailed information necessary for biomechanical analyses [11]. Some studies have explored the applicability of Microsoft Kinect as the only sensory input for the CL of an FES system [13,14]. In general, Kinect requires an RGB camera for image capture and an infrared camera for depth estimation to provide the three-dimensional coordinates of objects. Furthermore, using a sequence of images, it is possible to estimate the speed at which objects are moving. It also includes efficient algorithms that allow the detection of the body skeleton in real time. One of the advantages of Microsoft Kinect is that it allows the estimation of the distance and pattern of the moving objects in the scene [13]. Nevertheless, these studies were only tested for upper limb applications (assisted grasping).

Also, ultrasound (US) imaging-derived echogenicity signals have been used for measuring muscle activation [15]. Authors refer to US echogenicity as the ability to reflect US waves in the context of surrounding tissues, and it can be used to estimate its displacement. Echogenicity is obtained by calculating the average intensity of pixels, within a region of interest, in each frame of the generated images. However, due to the high computational cost of processing US images, the sampling rate allowed by this technique is low.

Another approach, which has apparently been less commonly applied for that purpose, is the use of binocular vision systems. A binocular vision system requires only two calibrated RGB cameras to estimate the three-dimensional distance between objects, via the triangulation of corresponding points in both cameras. The hardware required for this approach can be even cheaper than that required by depth cameras.

However, it remains unclear whether vision-based non-wearable technologies have been fully exploited beyond subjective assessment and documentation. The potential use of standard camera footage for motion analysis would require simpler setup requirements and fewer recording constraints, and could be integrated more seamlessly into daily research, clinical, and telemedical settings [11]. These characteristics could potentially result in lower costs, providing non-wearable marker-less tools with significant advantages over traditional gold-standard technologies, which are expensive and challenging to implement in everyday activities where FES systems are used and evaluated.

### 1.2. Control Strategies

Once human kinematics and muscle activity are measured, this information can be used to adjust the stimulation parameters automatically to the needs of the user in order to delay the onset of fatigue and to target desired movements in an optimal way. These needs can be addressed through the implementation of feedback control [10].

A variety of control strategies are utilized to replicate or support functions carried out by the central nervous system (CNS), enabling the activation of muscles for the execution of natural movements. These strategies can be classified into two modalities: open-loop (OL) or closed-loop (CL) [16].

An OL control strategy in FES is a straightforward yet dependable method for regulating stimulation timing. OL architectures are commonly utilized in medical environments. These systems offer less precise movement control as they depend on manual input to initiate stimulation delivery [8,17].

On the other hand, CL FES control strategies can enhance the stability and robustness of position and force control by adjusting various stimulation parameters. These strategies aim to correct model errors and address internal disturbances (such as muscle fatigue) and external disturbances (such as obstacles) through feedback information [16].

### 1.3. Objectives and Structure of the Paper

This SLR aims to identify which sensing and control methods are currently used in lower limb FES applications, and to carry out a systematic comparison of key features related to them, such as invasiveness, cost-effectiveness, ease of use, practicality and suitability for the target application.

This SLR can serve as a complement to other recent reviews [18] that explore control strategies and different type of sensors for upper and lower limb applications of FES. It is our goal to help researchers interested in FES by carrying out a process of selecting suitable combinations of sensing and control strategies for lower limb FES applications, taking into consideration technical and clinical parameters. For example making a more informed choice would help to implement more effective FES control systems for lower limbs. Once these variables are chosen, they directly influence the type of sensor. Although several papers have been published on the exploration of sensors and control strategies, many of them do not consider computer vision as a measurement tool for the controlled variable.

The main aim of this work is to perform a SLR of alternative measurement methods for parameters used in lower limb FES control systems, such as foot angle and velocity. A secondary aim is to explore current and potentially novel approaches for control and sensing in lower limb FES systems and applications, beyond classical approaches.

The remainder of this article is organized as follows. First, the Materials and Methods section details the search strategy and how the PRISMA methodology was followed to conduct this SLR. The analysis of each article reviewed is presented in a table format. In Section 3, the results are presented, addressing each of the research questions proposed in Section 2. Additionally, the main findings of the results are highlighted, suggesting a classification based on the analyzed data. Section 4 covers the discussion, where the implications of the findings of the SRL are mentioned and proposals are given for implementation in FES systems. Finally, a conclusion is given in Section 5.

## 2. Materials and Methods

The PRISMA methodology was followed to conduct the SLR search [19]. A set of four academic and scientific databases were searched, PubMed, Scopus, Wiley Online Library, and Web of Science, from 2010 to 2024. Records were imported to the web application rayyan.ai for filtering and classification. This SLR does not have registration information.

### 2.1. Search Strategy

**Figure 1:**
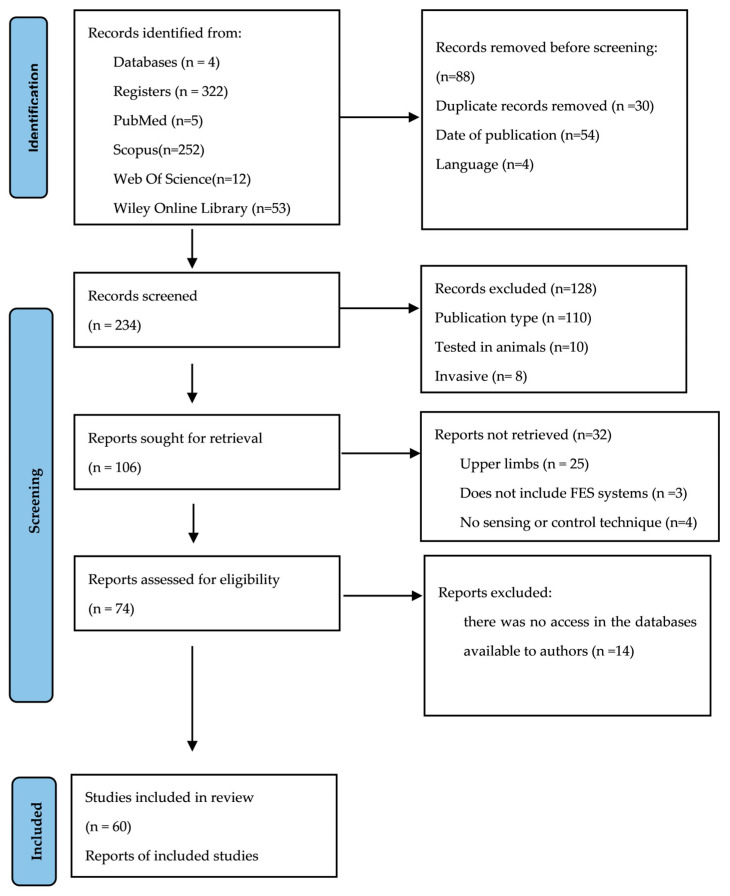
PRISMA flow diagram.

The three steps involved in the manual literature search process are summarized in the PRISMA flow diagram in Figure 1. In the first step (Step 1—Identification), the titles of articles reporting sensing and control strategies were identified from electronic databases. It was specified that the search terms should be found in the titles, keywords, and abstracts in databases that allowed such specification, such as Web of Science and Scopus. Data collection was performed independently by two authors to reduce the risk of selection errors and selection bias. The database search concluded in April 2024. Then, data extraction from abstracts and keywords was performed, and duplicate records, unrelated studies, and articles published before 2010 were removed.

The second step was a more detailed review of the full-text articles (according to the inclusion and exclusion criteria), to assess the eligibility of the selected papers (Step 2-Screening). If the abstract did not indicate clearly whether or not the inclusion and exclusion criteria were met, the full-text paper was also read. In the last step (Step 3-Included), studies considered relevant and those on recent advances were selected for further analysis in this SLR. The last step of filtering was applied to papers after reading the full text, taking into consideration whether or not they reported any sensing or control strategy.

#### 2.1.1. Types of Population

This SLR focuses on works where FES systems and interventions are designed or ap-plied for the assistance or rehabilitation of patients with FD. However, FES technology is particularly useful for the treatment of foot drop of central origin, when peripheral structures (nerves, muscles, and joints) are functional. Additionally, this SLR was not limited only to poststroke patients, in order to obtain a more comprehensive overview. Finally, the review included those studies that tested FES systems or interventions in healthy volunteers and that reported on the development or preliminary testing of FES systems for patients with FD.

#### 2.1.2. Types of Intervention

This review included studies that used FES systems for gait assistance or rehabilitation. Only studies where non-invasive, surface electrodes were used for the application of FES were included. Works using any type of invasive stimulation electrodes were excluded. Reports of hybrid systems that combined FES with another type of actuator such as exoskeletons were also included for the same purposes. Technical tests, proof-of concepts, pilot studies, and controlled clinical trials were included.

#### 2.1.3. Types of Comparison

Due to the technical nature of this SLR, no comparison was performed in clinical terms. Only the applications of different control and sensor strategies employed in the different reports were compared.

#### 2.1.4. Types of Outcomes

No outcomes were considered, since the purpose of the paper was to explore the application and combination of different control and sensing strategies in FES systems for FD assistance and rehabilitation.

### 2.2. Research Questions

As a complement to other reviews that explored control strategies and sensors for FES systems [18], our objective was to focus this research on drop foot FES applications and to expand the investigation to include vision systems as a new sensor. The aims of this SLR were translated into a set of research questions to better explain and summarize the evidence about FES systems for lower limb applications.

RQ1: What types of sensors are used to measure the body’s state and its response to FES?

RQ2: What variable is measured by this sensor?

RQ3: Which control strategies are employed in lower limb FES systems and applications?

RQ4: What stimulation parameters are modulated in CL lower limb FES applications?

### 2.3. Inclusion and Exclusion Criteria

The keywords used for a Boolean search through the databases were as follows: (“Foot Drop” OR “Drop Foot” OR “Dropped foot” OR “Foot drop syndrome”) AND (“Functional electrical stimulation” OR “Therapeutic Electrical Stimulation” OR “Electrotherapy” OR “Electric stimulation”) AND (“Electromyography” OR “EMG” OR “Electric Myography” OR “Elec-tromyographic techniques” OR “Inertial sensors” OR “Inertial sensing devices” OR “Inertial measurement units” OR “IMUs” OR “Computer vision” OR “Machine vision” OR “Vision systems” OR “Leap Motion Controller” OR “Kinect” OR “Stereoscopic vision” OR “ToF camera” OR “Structured light imaging”) AND (“Open-loop” OR “Open-loop control systems” OR “Open loop” OR “Closed-loop” OR “Feedback sensory” OR “Feedback mechanisms” OR “Closed-loop systems” OR “Closed loop”).

Articles in which the subjects of study were animal models were excluded. Also, articles using invasive electrodes and those that focused on upper limbs were excluded as they were not of interest for this SLR. SLRs and conferences older than 3 years were also excluded. Additionally, book chapters, abstracts and posters, and any articles written in languages different to English were excluded.

### 2.4. Quality Assessment

To analyze the information, all the 60 included articles were reviewed, to extract from them relevant data based on the questions asked and other information considered important, such as the test subjects, the type of target movement, the purpose of the study, and the type of application (rehabilitation or assistance). It is crucial to note that some articles did not report or focus on some of the parameters mentioned above.

### 2.5. Data Extraction and Analysis

To analyze the information, all the 60 included articles were reviewed, to extract from them relevant data based on the questions asked and other information considered important. It is crucial to note that some articles do not report or focus on some of the parameters mentioned above. Experimentally informed studies and the data acquisition reported in each article were analyzed. This analysis aimed to evaluate the type of movement, the sensor used, and the variable being measured. It also sought to establish the health condition of the participants in each study. With regard to the sensing strategy, the equipment (sensors) utilized for data acquisition, as well as the specific parameters that were measured during this acquisition, was considered. Subsequently, the control strategy pursued by each article was examined. It should be noted that some articles did not report on the implementation of a control strategy, while others did not provide sufficient detail regarding the strategy’s implementation. It is also important to mention that, in some cases, the control strategy was not applied to the users from whom the data were obtained but was instead implemented in a simulation. Also, due to its relevance to the FES control strategy, an additional analysis was conducted to examine whether or not and how the electrical parameters were modulated.

## 3. Results

The 60 papers remaining after the filtering stages, out of the original 322, were included as relevant to this SLR and then selected for data extraction and further analysis.

Table 1 shows the 60 articles considered relevant for this SLR.

It is important to mention that in 45% (27) of the articles, tests were conducted with healthy subjects. In total, 35% (21) performed tests with patients, while 16.7% (10) conducted tests with both patients and healthy subjects. Also, it was observed that 31.7% (19) of the tests were conducted with subjects seated, another 38.3% (23) were performed while subjects were walking, and 11.7% (7) performed both types of tests. For 78.3% (47) of the analyzed articles, the goal of the work was rehabilitation, while 11.3% stated that the purpose was assistance. The remaining works considered both rehabilitation and assistance as objectives.

It is worth highlighting that the target population in most articles was stroke patients (19). Other pathologies found in the included articles were spinal cord injury (SCI) (15), neurologically impaired individuals (8), and CNS disorders (3). Some studies did not specify the pathology they focused on (13). The remaining studies focused on pathologies that were mentioned only once.

### 3.1. RQ1: What Types of Sensors Are Used to Measure the Body’s State and Its Response to FES?

For the analyzed articles, sensors were classified according to their purpose into four categories: switches, electrodes, vision-based, and IMUs. Additionally, it is important to note that most papers utilized more than one type of sensor.

In total, 45% (27) of the articles used IMUs as sensors to measure body/limb posture. About 36.7% (22) used electrodes, encompassing terms such as “EMG sensors”, “sEMG”, “Electrode pads”, and “EEG sensors”. Additionally, the category of vision-based systems was included, which comprised all systems using images generated by any hardware. This category was present in 18.3% (11) of the articles. The systems included in this category are as follows: motion capture systems, 11.7% (7); US imaging, 5% (3); and depth camera, 1.7% (1). Meanwhile, 18.3% (11) were categorized under switches, including terms like “FSRs”, “forefoot switches”, “heel switches”, “force transducers”, “Foot pressure insoles”, and “Foot Plantar Pressure Sensors”. Goniometers were present in 6.7% (4), while dynamometers also were present in 5% (3) of the articles.

As shown in Table 1, there were other types of sensors used for these measurements. However, it is relevant to mention those that are more commonly used. Other sensors were analyzed in the category of others, 28.3% (17).

**Figure 2:**
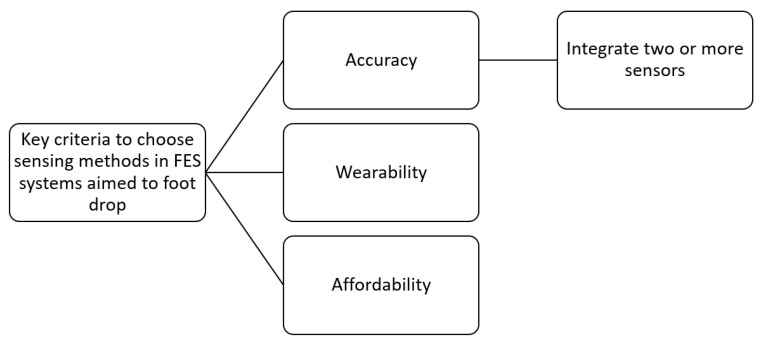
Key criteria to choose sensing methods in FES systems for FD.

The results yielded a set of key criteria (Figure 2) useful for selecting sensor strategies in FES systems targeting FD. It is important to note that the inclusion of more than one of these criteria is essential for consideration in an FES system. However, articles were divided according to the main criterion they considered in order to categorize them. It is also important to mention that this categorization is based solely on the sensing method. Those articles that did not focus on the criteria for selecting the sensor were not included in this categorization. The proposed criteria are described in detail below and illustrated in Figure 2.

- Accuracy

Twenty-three of the articles included in this SLR emphasize the necessity of employing a sensor or sensing strategy that allows for precise measurement. The design of systems for gait rehabilitation is a complex process that requires precise and robust control systems that incorporate sophisticated actuation and sensing capabilities. Precise identification of gait phases and a responsive control strategy are vital for effective assistance and rehabilitation of walking using FES, since they directly affect the precise timing of stimulation. To achieve suitable controllers that deliver stimulation that mirrors normal muscle activation patterns, reliable online detection of gait phases has been shown to be essential [17,33,43]. It is of great importance to acknowledge that the accuracy of a FES system is reliant on both the selection of the sensor and the processing and control algorithm technique employed. However, the focus of this section is the examination of the precision of the sensors utilized.

In [38], the authors address that a FD stimulator should be able to differentiate between walking and other type of activities (exercises) to avoid unnecessary stimulations in non-walking conditions. This allows patients to safely warm up with the device donned and eliminates the need to remove it after warming up.

Another example where accuracy in some aspect of FES systems has been shown to be a factor in the performance of FES systems for gait assistance and rehabilitation is the work reported in [17]. The researchers created a real-time gait phase detection system for an FES system, with optimized sensors and data processing. However, they found that the use of wearable IMU sensors caused a delay in the detection of gait phases, and led to stimulation timing errors, which may have increased the time shift between the intended and actual actions assisted by FES by up to 100%. The origin of those delays can come from wireless IMU data streaming, USB communication protocol timing uncertainties, or even the non-real-time nature of the Windows operating system.

In order to improve accuracy in FES systems, multiple studies, 46.7% (28), included in the SLR used two or more sensors. Previous research [74] indicates that a single foot switch, due to its low detection reliability, is not suitable for triggering FES stimulation sequences for walking assistance/rehabilitation. Therefore, the researchers developed a system comprising a novel sensor combination: three FSRs to measure the load force in a shoe insole and a miniature gyroscope chip to measure the foot’s rotational velocity. This way, the authors achieved a novel gait phase detection sensor that accurately identifies transitions between the stance, heel off, swing, and heel strike phases of walking.

In another work [21], the authors reported that using only motion sensors in FES systems poses challenges to their performance, due to their low signal-to-noise ratio and the need for extensive signal post-processing. Therefore, they report on the combination of FSRs and motion sensors. By placing FSRs under the foot and attaching accelerometers to the shank, feedback signals can be gathered to generate FES sequences for four muscles, guided by a controller with rules derived from human data.

- Wearability

The authors of 18 of the articles presented in this SLR were motivated to implement wearable sensors, although these studies also sought to achieve high accuracy. The primary considerations influencing the selection of sensors were the practicality of incorporating them into daily activities or settings outside the laboratory, and the ability to allow patients to walk freely. Sensors in FES systems are often employed to measure the state of the body through variables such as muscle force, joint angle, velocity, and acceleration. Although some variables are measured with specific sensors, sometimes they are not practical for the operation conditions of the system. Some projects involving FES for FD are suitable to be used solely in the laboratory, while others are operated outside of it. For ex-ample, since torque is considered an important variable related to bodily response [68] in FES systems, the use of conventional, mechanical torque sensors is desired. However, torque sensors that are available commercially are unsuitable to be used in patients’ daily lives. Similar reasons have motivated researchers to employ different types of sensors to indirectly measure some variables; for example, a number of methods have been proposed to estimate muscle force or joint torque from sEMG signals [46,75].

Another situation related to wearability is when multiple stimulation electrodes and sensors are placed on the body, which is complicated when using traditional wired connections. Wireless sensing and stimulation systems offer a more streamlined and mobile solution to their wired alternatives. However, only a few external wireless FES stimulators currently attempt to connect and coordinate individual channels via a network. In this regard, in [66], the authors presented a wireless distributed FES architecture. The system relies on the collection of potentially diverse distributed stimulation and measurement units overseen by a wearable controller.

- Affordability

While less predominant, five of the articles that addressed the motivation behind the selection of sensors were driven by the desire to implement sensors that are cost-effective. In some instances, this objective coincides with that of the incorporation of wearable sensors.

The rise in the use of open-source and low-cost prototyping electronics has enabled the popularization of platforms like the Arduino microcontroller unit to be employed in the development of a wide variety of electronic systems. These platforms utilize high-level libraries that allow for the quick and easy integration of various sensors and actuators into a testing platform, making them ideal for developing low-cost, portable gait neuroprostheses. In [22], a flexible, low-cost micro-controller-based platform for the rapid prototyping of FES-based neuroprostheses is presented. The system was designed to reduce computational complexity, the time for development, and production costs. Its small size and weight (225 g with all modules connected) make it perfect to be attached to a below-the-knee cuff or at the hip.

The study conducted by [73] sought to explore the practicality of using a more cost-effective and accessible sensor, such as a depth camera or pressure mat, as an alternative to the force plate.

### 3.2. RQ2: What Variables Does the Sensor Measure in Gait-Oriented FES Systems?

Due to the dispersion of the variables, we decided to categorize them for the analysis. The category *Angles*, which includes all variables related to angles of body segments, such as knee joint, ankle joint, foot, etc., was present in 38.3% (23) of the articles.

Another category included was electrical activity, which was present in 18.3% (11) of the articles included in this review.

The next most used variable was angular velocity, which appeared in 13.3% (8) of the articles. Following this, acceleration was present in 11.7% (7) of the works. Another category that was found to be important to group was *Torque*, which encompassed all methods related to torque measurements from different parts of the body; this category appeared in 10% (6) of the articles. Finally, the category gait phases, which includes all variables related to identifying gait features, such as heel strike and toe off, gait events, etc, was present in 10% (6) of the articles. The remaining variables were reported only in one or two works and can be seen in Table 1. Some of them are commented below.

In [30], the researchers carried out preliminary work on a sensor-based multichannel FES system for post-stroke gait where FES was used to control knee joints. Their research highlights that the application of FES to a stiff knee in hemiplegic individuals reduces spasticity in both the knee flexors and extensors, while enhancing their range of motion. Additionally, they highlight that avoiding hyperextension and allowing slight knee flexion in the affected limb during the stance phase significantly aid in gait recovery. Therefore, accurate control of knee angle is vital for improving gait in people with CNS disorders.

Due to FD, patients often experience ankle dyskinesia. In [45], the human ankle dorsiflexion angle is considered the research object in the authors’ FES system. An accurate ankle model helps explore movement characteristics under different electrical stimulation parameters. The motion characteristics of the ankle angle, including hysteresis, time variance, and nonlinearity, can be summarized based on experiments that measure ankle angle changes induced by electrical stimulation.

In [58], the researchers predict torque output based on evoked electromyography (eEMG) recordings only. Volitional EMG-based joint movement estimation is effective in healthy subjects; however, SCI patients cannot generate volitional EMG due to spinal cord damage. Consequently, performance prediction is more stable in healthy subjects than in SCI patients. On the other hand, evoked signals in SCI patients are reliable due to the absence of volitional contractions, since their weaker muscles result in less progressive and stable recruitment. Nevertheless, the extent of spinal lesions varies widely among SCI patients, requiring more patient-specific adjustments compared to healthy individuals.

### 3.3. RQ3: Which Control Strategies Are Employed in Lower Limb FES Systems and Applications?

In total, 63.3% (38) of the articles employed a CL strategy, 8.3% (5) employed an OL strategy, and the remaining were categorized as Others. Some articles reported the strategy used to implement the CL, with the most common being finite state machine, 8.3% (5); iterative learning control, 5% (3); and proportional–integral derivative (PID), 1.7% (1); and P controller, 3.3% (2).

According to some works in this SLR, adaptive CL control is ideal for FES systems but is rarely developed [17,43]. Most commercial FES-based gait rehabilitation systems use simpler OL control solutions. Parastep I (Sigmedics, Inc., Fairborn, OH, USA) and RehaStim (Hasomed Inc., Magdeburg, Germany) are two commercially available FES systems that use OL control strategies. These simple solutions demand continuous or repeated user input, usually via a device button, for muscle activation, thus requiring the complete focus of the user, clinical personnel, and/or caregiver [17,43].

Finite-state-controlled FES systems combine the accuracy of CL controllers with the simplicity of OL setups, using minimal sensors, generally single or a few foot switches, or an IMU. In general, they operate with preset stimulation sequences triggered by specific conditions. However, there are works that report offline adjustments between the period of use as a strategy to combat muscle fatigue [17].

Additionally, six articles were found that reported on the use of machine learning techniques. Among them, neural networks were the most used technique, with a focus on model accuracy and personalization to improve clinical outcomes. As examples, three studies are described below.

In [45], ankle angle characteristics are used to train a Hammerstein (H) model based on a neural network, with model parameters identified using a genetic algorithm. This approach effectively predicts ankle angle changes induced by electrical stimulation. Experimental results confirm that the neural-network-based H model can accurately forecast the output changes in the ankle angle due to the electrical stimulation pulse.

Another study [58] introduces a real-time system for estimating FES-induced torque, utilizing a wireless portable stimulator. The system uses a Kalman filter and recurrent neural network (RNN) to predict torque output from eEMG recordings. Experiments with able-bodied subjects and SCI patients demonstrate its promising performance. This system offers personalized muscle response evaluation, beneficial for clinical diagnostics.

Another paper [60] introduces a new machine learning approach to improve lower limb tracking control for individuals with SCI through neuromuscular electrical stimulation/FES. The method uses data-driven models with historical rehabilitation data, applies robust integral of the sign of the error (RISE) as a control technique for stability, and employs an enhanced genetic algorithm for effective controller tuning.

Reinforcement learning (RL) is a machine learning technique that relies on rewards and involves an agent who observes the environment and learns an optimal policy for action selection based on the states [26]. In [26], the researchers introduce an RL algorithm utilizing a decayed epsilon greedy approach to investigate different pulse parameter variations, with the goal of optimizing stimulation patterns during FES cycling sessions.

### 3.4. RQ4: What Stimulation Parameters Are Modulated in CL Lower Limb FES Applications?

In total, 60% (36) of the articles specify the use of adaptive FES systems. Some of them specify which parameters are modulated, with the most frequently modulated ones being pulse width, 26.7% (16); intensity, 8.3% (5); timing, 8.3% (5); frequency, 3.3% (2); and amplitude, 8.3% (5). Below, three examples of parameter modulation strategies are described.

In [45], an experimental platform was used to study the effects of different electrical stimulation parameters on ankle motion characteristics. The parameters investigated were frequency, amplitude, and pulse width. The findings highlight that lower frequencies are preferred to avoid muscle fatigue, with 25 Hz being optimal. Increased amplitude generally led to a greater ankle angle, with 25 mA being the ideal setting. Adjustments in pulse width affected the ankle angle, with discomfort noted at higher widths. The results provided valuable data for neural network-based model input parameters and demonstrated that electrical stimulation parameters significantly influence ankle joint motion.

In 2016, the authors of [27] proposed a control method for an FES system aiding in walking. It adjusts the quadricep stimulation amplitude based on knee flexion angles increasing it by 10 mA for angles over 10° (knee unlock), maintaining amplitude for 5–10° (knee extension), and gradually reducing it by 5 mA over 2 s for angles below 5° (knee lock). After each increment, a 1 s interval follows before potentially adjusting it again, with flexibility if knee buckling exceeds 10° per second. This approach aims to optimize muscle stimulation for enhanced stability and control during the stance phase of walking, adapting in real-time to knee angle feedback.

In the work reported in [33], the aim was to optimize FES assistance by dynamically adjusting stimulation parameters according to real-time feedback, thereby improving the effectiveness of gait assistance across different walking speeds and conditions. The parameters (τ and PW) were updated dynamically based on data from the previous five gait cycles, ensuring that the assistance provided by the FES system aligned with the current walking conditions and speed.

## 4. Discussion

### 4.1. Sensing

Feedback signals based on artificial or natural sensors are required to build CL control systems for FES-based gait assistance.

Although sensors are employed to measure the state of the body, response variables such as muscle force, joint angle, velocity, and acceleration are not the only factors to con-sider when choosing which sensor or sensors to use in the FES system. Even though certain variables may seem more suitable to measure with specific sensors, this is not the sole consideration.

A key consideration is the purpose for which the FES system will be implemented. Some projects are intended to be used solely in the laboratory, while others aim to be utilized outside of it. For example, the implementation of torque sensors is often pursued because torque is considered an important bodily response. However, existing torque sensors are unsuitable to be used in patients’ daily lives. Reasons like this motivate the search for non-evident or conventional sensors to indirectly measure some variables. Other examples include reports of methods that have been proposed to estimate muscle force or joint torque from sEMG signals [46,75].

Another consideration is the choice of the control system. Choosing a sensor digitized with a low sampling rate can be challenging to incorporate into CL FES control. For instance, the sampling frequency of the echogenicity signal derived from US imaging is significantly lower than kinematic measurements obtained from IMUs or angular encoders [15]. This serves as an example that some reports of the implementation of FES systems employing novel sensing strategies are not always aimed at demonstrating improvements over an existing method but rather at showcasing the potential advantages one sensor has over others.

Moreover, the performance assessment of FES systems is a crucial factor for their design. Previous research has concentrated on the torque or angle between intended and actual limb movements. For instance, Sharma et al. explored the nonlinear FES tracking control of human limbs and assessed the system used by monitoring the knee’s trajectory to control the angular deviation from the ideal path. Zhang et al. (2013) employed sEMG-based CL torque control in the FES system and analyzed the discrepancy between the generated and desired torque [32].

Furthermore, it is important to mention that some sensors are more complex and expensive than others; this is another important thing to consider. For example, the use of motion capture data is another common sensing strategy utilized for feedback and the assessment of FES systems’ performance; however, the equipment used for capturing gait using video (photogrammetry) is costly and complicated. A video-based motion capture system typically involves placing reflective markers on the subject’s skin or using specialized clothing to record human movement using cameras and specialized software. Additionally, the captured video image data can be easily influenced by factors such as lighting and range of movement [73].

According to the articles reviewed, IMUs and biopotential electrodes are the most commonly used sensors in research. It is also important to mention that most of the analyzed articles use more than one sensor.

Taking all of this into account, to aid in the development of a more robust FES system, it is important to explore new alternatives that have high measurement precision, are portable, can be integrated into control systems, and are not prohibitively expensive.

To our knowledge, and based on the results of this SLR, few articles have used depth cameras as a method of measuring the body’s response to FES. In fact, only a few articles were found in this review to employ depth cameras, and these were mostly focused on balance training or upper limb applications [73,76].

Using depth cameras as feedback sensors, cheaper, and more portable FES systems can be implemented. In 2024 [73], a depth camera showed higher potential as a low-cost, portable sensor, compared to a pressure mat in an FES balance application. Also, a depth camera is a feasible option as a replacement for the force plate for use in a FES + VFBT (virtual feedback balance training) system.

Furthermore, no works were found that used binocular vision, which can lead to similar results to those of using depth cameras, at a lower cost. This is because infrared sensors used in depth cameras are more expensive than RGB cameras. The above discussion opens up an area of opportunity to explore new techniques and improve existing ones.

Measuring the body’s state and its reactions can provide valuable information about the system’s effectiveness, with each type of measurement offering different insights. For example, FES-restored motion relies on active joint torques and environmental interactions. Joint angle feedback cannot distinguish between motion from stimulation and external forces, making joint torque control superior. Torque control provides essential compliance, especially for environmental interactions and daily human activities [46].

Focusing on a specific joint can be crucial for post-stroke gait recovery. Several studies have indicated that preventing hyperextension and allowing slight knee flexion of the paretic limb during the stance phase can significantly enhance gait recovery. This is the reason why many studies focus on measuring the knee joint [30].

### 4.2. Control Strategies

When it comes to muscle stimulation, most commercial FES systems operate in OL mode, where the stimulation intensity—comprising pulse width and pulse amplitude—remains constant. The primary drawback of OL FES is its inability to adjust stimulation intensity in response to changes in residual muscle activity and spasticity, which can happen within as few as five strides [34].

In contrast, a CL FES control strategy is considered a better solution for gait rehabilitation applications, as it more accurately replicates the nervous system’s control of gait. This approach uses feedback information to modify stimulation parameters according to the desired joint angle or moment trajectories [43]. The difficulties in CL control stem from the complex and nonlinear nature of electrical muscle stimulation, involving unpredictable mapping from electrical input to muscle force, muscle fatigue, and delayed muscle responses [39]. To address these challenges, various control strategies have been sought that are robust against unpredictable changes in input delay and the uncertain dynamics of muscles.

The key muscles involved in gait include the hamstrings (biceps femoris), quadriceps (vastus medialis), calf muscles (soleus), and tibialis anterior; each have unique functions during the stance and swing phases of the gait cycle, and they are activated in a specific order [77]. In FES-based rehabilitative gait systems, the activity of these muscles must transition in the same sequence. A finite-state machine (FSM) can be employed to achieve this, as it allows for the sequential stimulation of muscles. An FSM model, which sequences ON/OFF actions, can control the timing of muscle stimulations within the different states of the gait cycle [43].

Another strategy used is iterative learning control (ILC), which is suitable for systems performing repeated tasks [4]. ILC aims to minimize trajectory tracking errors through repeated system operation. It continuously adjusts the controlled system’s trajectory towards the desired path, characterized by a strong mathematical foundation and flexibility for systems with uncertain parameters, which are particularly beneficial for nonlinear systems [52].

The functional effectiveness of an FES system for gait heavily depends on the precise timing of the applied stimulation within the gait cycle. The most straightforward way to control this timing is through manual press buttons or foot switches, which constitute a common approach in most commercial products [21]. The success of FES in treating FD patients relies on the control and modulation of stimulation intensity. Two popular modulation methods for FD intervention are the trapezoidal stimulation profile and the EMG-modulated natural stimulation profile [32].

Although the use of machine learning-based control algorithms was reported in some works reviewed in this SLR, deep learning algorithms were not found, although these techniques have been applied in other technologies used for motor rehabilitation that require interaction with the user, like brain–computer interfaces [78]. In the future, it would be interesting to attempt to incorporate such algorithms to improve sensing/control strategies, for example, to create intelligent–adaptive FES systems, that require no or little previous calibration.

The integration of these kinds of intelligent algorithms, trained with large databases comprising multimodal variables from conventional and novel sensors, validated by gold-standard techniques, such as photogrammetry, would contribute greatly to improving the design and performance of FES systems. To this end, multi-center collaboration would be ideal in overcoming the limitations of the sample sizes of most works, especially when treating patients with FD caused by low-incidence medical conditions, like spinal cord injury or cerebral palsy.

One of the main limitations identified in the studies reviewed is that they tested their systems only with healthy volunteers or with a small sample of patients. Hence, larger pilot and controlled clinical trials are required to better assess the benefits and feasibility of novel sensing and control approaches. Another key limitation was that many studies did not explicitly report the type of sensors they employed, which reduced the information available.

To summarize, some key considerations for practical applications derived from this SLR are as follows:

- Applying a multimodal sensing strategy, comprising more than one sensor, is a highly recommended approach in CL gait-oriented FES systems for FD, with the potential to improve system accuracy and performance.
- Novel sensing and control strategies, such as US sensors and machine/deep learning-based controllers, should be considered, possibly combined with the multimodal approach mentioned above. To achieve this, fast and reliable digitizing and processing systems are required, to be feasible in real-life applications.
- The integration of markerless motion analysis tools, employing computer vision and depth sensors, is an alternative approach worthwhile to explore in gait-oriented FES applications, the relevance of which has already been shown in upper limb and stability training applications.

## 5. Conclusions

This SLR will serve as a global framework of sensing and control strategies employed in FES systems for gait rehabilitation for patients with FD. This topic is of great relevance to researchers and developers, since both elements play a fundamental role in the overall performance of FES systems and applications; knowing beforehand the different existing techniques already implemented and tested by other research groups could be vital at the initial stages of the design process of an effective FES system, according to specific needs and purposes.

Taking into account the criteria proposed for the selection of sensors for FES systems, one conclusion is that the use of depth cameras as a measurement method is feasible, these being non-invasive, portable, and low-cost sensors. Future research will focus on comparing the accuracy of depth cameras and other vision-based methods with other sensors mentioned in this SLR.

In particular, the variable angles was of utmost interest in the articles analyzed, due to its direct relation with the target ankle movement to be induced by the FES system. Furthermore, it is worth noting that measuring the angle variable through depth cameras is highly feasible, in static or dynamic conditions, through signal processing algorithms.

For future research, it is recommended to further explore the implementation of control methods based on machine learning algorithms, especially in combination with multimodal sensing techniques if possible, including novel approaches in the field, such as US or depth cameras/computer vision. Such combinations could allow the development of better, more natural interfaces for FES systems, especially if they are coupled with high-performance computing systems, like Field programmable gate array circuits, real-time microprocessor devices, or even emerging artificial intelligence dedicated processing units.

## Figures and Tables

**Table 1:** Sensing and control strategies in FES systems.

Author	Subjects	Type of Movement	Purpose	Sensing Technology/Strategy	Measured Variable/Signal	Control Strategy	Parameters Modulated
[20]	Healthy	Sitting	Rehabilitation	EMG and EEG sensors Torque transducers		Real-time control interface of the MAFO	-
[21]	Healthy	Walking	Assistance	FSRs IMUs	Ground reaction force Angular rate Acceleration	Reflexive control algorithm	Timing and coordination of muscle activations Stimulation sequences for specific muscles’ stimulation amplitude
[22]	Healthy	Walking	Rehabilitation	IMUs FSRs	Dynamics of the leg and foot Gait events Ground reaction forces	CL	Pulse width
[23]	Both	Sitting	Rehabilitation and Assistance	Load cell	Torque generated by the muscle contractions Knee joint angle	CL, PID	-
[24]	-	Sit-to-stand	Assistance	-	-	Combination of sliding mode control and wavelet networks	-
[25]	Patients	FES cycling	Rehabilitation	IMUs	Angular velocity Linear acceleration	Adaptive method	Timing of muscle contractions
[26]	Healthy and patient	Walking and sitting	Rehabilitation	sEMG sensors Torque sensors Kinematic sensors	Joint angles Angular velocity Acceleration	Linear control schemes	Stimulation intensity Pulse width Frequency
[27]	Patients	Walking	Rehabilitation	IMUs	Angular displacements	CL, FSM	Stimulation amplitude
[28]	Healthy	Sitting	Rehabilitation and Assistance	Force transducers	Force	CL	Stimulation amplitude
[29]	Patients	Walking/sitting	Rehabilitation	EMG/EEG		-	-
[30]	Patients	Walking	Rehabilitation	IMUs Force sensors	Knee angle Gait events	CL, Proportional controller	Pulse width
[31]	Healthy	Walking and sitting	Rehabilitation	Motion capture system	Ankle joint angle	CL, ILC	Pulse width Stimulation intensity
[32]	Both	Walking	Rehabilitation	sEMG sensors Pressure Sensors	Angular velocity Ground reaction forces	CL, NS	Stimulation intensity
[33]	Healthy	Walking	Assistance	FSRs IMUs	Sagittal shank angle Angular velocity Acceleration	CL, FSM	Time coefficient parameter (τ) and pulse width
[34]	Patients	Walking	Rehabilitation	IMUs	Angular velocity Acceleration	CL, Proportional controller	Pulse width
[35]	Healthy	Sitting	Rehabilitation	Goniometer	Knee joint	Musculoskeletal model-integrated iterative learning control (MMILC)	Pulse width
[36]	Patients	Walking	Rehabilitation	IMUs	Foot pose initiation of the subject’s step	CL, NS	Yes, NS
[37]	Healthy	Sitting	Rehabilitation	sEMG sensors	Volitional electrical activity of the muscles	CL, Gram–Schmidt filtering algorithm	Waveforms proportional to the measured vEMG envelope
[38]	Healthy	Walking	Rehabilitation	IMUs FSRs	Heel pressure, shank tilt, and foot rotations	CL, FSM	Yes, NS
[39]	Healthy	Sitting	Rehabilitation	Optical encoders	Knee joint angle	CL	Pulse amplitude
[40]	Patients	Walking and sitting	Rehabilitation and Assistance	sEMG sensors IMUs	Angles of the ankles and feet Electrical activity of muscles	CL, NS	PWM signals
[41]	Patient	Walking and sitting	Rehabilitation	IMUs	Foot pitch Roll rate state	CL, NS	-
[42]	Healthy	Sitting	Rehabilitation	IMUs	knee joint angle	CL fuzzy controller	Yes, NS
[43]	Healthy	Walking	Rehabilitation	sEMG sensors Ground reaction forces Motion capture	Electrical activity Timing of heel-strikes hip and knee joint	CL, FSM	Timing and sequence
[44]	Patients	Walking	Rehabilitation	Motion Analysis system	Muscle Forces Step length Ankle angles	OL	No
[45]	Healthy	Sitting	Rehabilitation	IMUs	ankle angle	neural network-based H model	Pulse amplitude
[17]	Healthy	Walking	Rehabilitation	IMUs Motion Capture System	Angular velocity	FSM	No
[46]	Healthy	Sitting	Rehabilitation	sEMG sensors	joint torque	CL, NS	Yes, NS
[7]	Patients	Walking	Assistance	IMUs	Foot angle and angular velocity	CL, NS	Pulse width
[47]	Patients	Walking	Rehabilitation	Forefoot switch accelerometer	Timing and intensity of muscle contractions		No
[48]	Both		Rehabilitation	Video-based motion capture/ IMUs/ Force-sensing resistance sensors/ EEG-sEMG sensors	Motion law of joints/ joint acceleration, velocity and angular acceleration/ planta pressure/ bioelectrical information	-	-
[3]	Healthy	Sitting	Rehabilitation	Encoder sEMG sensors	Knee joint angle, angular velocity, and acceleration	OL	-
[49]	Patients	Swiming	Assistance	IMUs	Trunk roll angle and rate Upper-arm inclination angle Knee joint angle Torso roll angle	CL and OL	-
[50]	Healthy	Sitting	Rehabilitation and Assistance	Leg extension machine	Torque produced about the knee joint	CL nonlinear control	Pulse width
[51]	Patients	Walking and sitting	Rehabilitation	IMUs	Accelerations, Angular rates of the foot	CL, ILC	Stimulation intensity and timing
[52]	Patients	Walking	Rehabilitation	angle sensors	angles of the knee joint	CL, ILC	Pulse sequences
[53]	Patients	Walking	Rehabilitation	Heel switches, accelerometers, and tilt sensors	Gait symmetry, rhythmicity, and ankle movements	CL, NS	Duration and intensity of the electrical stimulation
[54]	Both	Cycling	Rehabilitation	sEMG Optical encoder	Electrical activity Crank position	CL repetitive learning control	Pulse width
[55]	Patients	Sitting	Rehabilitation	Goniometers Dynamometers sEMG sensors		-	-
[56]	Healthy	Sitting	Rehabilitation	sEMG sensors	Motor-evoked potentials (MEPs)	OL	No
[4]	Patients	Walking	Rehabilitation and Assistance	Electrode pads	Angle of ankle dorsiflexion Position Electrical activity of muscles	CL, RC	Pulse width
[57]	Healthy	Walking	Rehabilitation	sEMG sensors	M-wave	CL model predictive control (MPC)	Pulse width
[58]	Both	Sitting	Rehabilitation	sEMG sensors Dynamometer	Evoked electromyography Ankle joint torque	-	-
[59]	Both	Sitting	Assistance	EMG amplifiers IMUs goniometers	Muscle activation patterns, Joint angles	-	-
[60]	Both	Standing	Rehabilitation	Electrogoniometer Gyroscope Accelerometers	Angular position of lower limb	CL RISE control strategy	-
[61]	Healthy	Sitting	Rehabilitation	US imaging	Delayed muscle activation	CL, NS	-
[62]	Healthy	Standing	Rehabilitation	force transducers optical encoders	torque produced about the knee-joint and the knee-joint angle	CL, asynchronous stimulation controller	Pulse width Stimulation channels
[63]	Healthy	Sitting	Rehabilitation	US	US echogenicity signal	CL, NS	Pulse width threshold and saturation values
[15]	Healthy	Walking	Rehabilitation	US Force plates IMUs	US echogenicity Ground reaction force 2D motion in the sagittal plane	CL, NS	Pulse width
[64]	Healthy	Squat/heel lift/walking	Rehabilitation	sEMG sensors IMUs	Motion intention Joint angle	CL, NS	Yes, NS
[65]	Healthy	Sitting	Rehabilitation and Assistance	sEMG sensors	Muscle activity	-	-
[66]	Patients	Walking	Rehabilitation	Distributed Measurement Units (DMU)	Knee joint angle Gait phases	CL, NS	Yes, NS
[44]	Patients	Walking	Rehabilitation	Foot switch Motion capture	Heel strike and toe off Muscle forces Step length Maximum knee and ankle angles	OL	-
[67]	Patient	Walking and sitting	Rehabilitation	Surface electrodes IMUs	EEG and EMG Tapping frequency of finger or foot	CL	-
[68]	-	-	Rehabilitation	EMG	Evoked EMG	CL	-
[69]	Simulation	-	Rehabilitation	-	-	OL	-
[70]	Healthy	Walking	Rehabilitation	Motion analyzer	Shank and thigh movement	Fuzzy controller	Stimulation intensity
[71]	Both	Walking and sitting	Assisted	Dynamometer	Torque	RISE controller	-
[72]	Patient	Walking	Rehabilitation	IMUs Force sensor	Posture information of limb movement Specific movement of limb	OL	-
[73]	Healthy	Standing balance exercise	Rehabilitation	Depth camera Pressure mat	Center of mass Center of pressure	-	-

EMG—electromyography; EEG—electroencephalogram; IMUs—inertial measurement units; FSRs—force-sensing resistor; CL—closed-loop; FSM—finite state machine; RC—reflexive control; OL—open-loop; NS—non-specified; ILC—iterative learning control; US—ultrasound; MAFO—motorized ankle–foot orthosis; RISE—robust integral of the sign of the error; PID—proportional–integral derivative; vEMG—volitional electromyography; sEMG—surface electromyography; PMW—pulse width modulation.

## Data Availability

No new data were created or analyzed in this study. Data sharing is not applicable to this article.
